# Safety, Pharmacokinetics, and Bioequivalence Characterization of Two Different Strengths of Mesalazine Gastro-Resistant Tablets

**DOI:** 10.3390/ph18121876

**Published:** 2025-12-11

**Authors:** Dolores Ochoa Mazarro, Manuel Román Martínez, Samuel Martín Vílchez, Sergio Luquero-Bueno, Paola Camargo-Mamani, Mariana Frau Usoz, Cristina Martínez Ostalé, Paula Arranz, Inmaculada Gilaberte

**Affiliations:** 1 Clinical Pharmacology Department, Hospital Universitario de La Princesa, 28006 Madrid, Spain; dochoa@iis-princesa.org (D.O.M.); manuel.roman@salud.madrid.org (M.R.M.); smvilchez@salud.madrid.org (S.M.V.); sergio.luquero@salud.madrid.org (S.L.-B.); 2 Clinical Research Department, Faes Farma S.A., 48940 Leioa, Spain; pcamargo@faes.es (P.C.-M.); mfrau@faes.es (M.F.U.); parranz@faes.es (P.A.); igilaberte@faes.es (I.G.)

**Keywords:** mesalazine, phase I, bioequivalence, healthy participants, ulcerative colitis

## Abstract

**Background/Objectives**: Ulcerative colitis (UC), a chronic inflammatory bowel disease, affects approximately 5 million individuals worldwide, exerting a considerable influence on global health and economic systems. Among the challenges in UC management, treatment non-adherence stands out as a critical issue, often compromising therapeutic efficacy. One strategy to address this challenge is by reducing pill burden, which may improve patient compliance and optimize treatment outcomes. **Methods**: This randomized, two-sequence, four-period, crossover replicate study evaluated the pharmacokinetic profiles, bioequivalence, and safety of a newly developed 1500 mg mesalazine gastro-resistant tablet compared to three of the reference 500 mg Claversal^®^ gastro-resistant tablets (total dose 1500 mg) in 80 healthy participants under fasted conditions. **Results**: Bioequivalence between mesalazine formulations was observed in both the rate and extent of systemic bioavailability. The geometric mean ratios and their 90% CI were 102.51% (95.85–109.63) for AUC_0–∞_, 103.36% (96.40–110.83) for AUC_0–t_, 84.49% (78.24–91.24) for AUC_8–48h_, and 114.24% (100.15–130.32) for C_max_. All within the accepted bioequivalence ranges, confirming comparable pharmacokinetic performance. Secondary pharmacokinetic parameters such as t_max_, t_1/2_, K_e_, Cl, and MRT were also consistent across both formulations. The incidence of adverse events was comparable between the two mesalazine formulations, with only flatulence and mild self-limited rash considered possibly related to test treatment. **Conclusions**: Overall, the 1500 mg formulation demonstrated a pharmacokinetic profile and tolerability comparable to the reference formulation, offering a higher-strength option to reduce daily pill burden. This strategy is of clinical relevance, particularly for improving treatment adherence among UC patients who need to take multiple pills daily to achieve their required dosage. While adherence is influenced by various factors, reducing pill burden may facilitate compliance and optimize therapeutic efficacy.

## 1. Introduction

Ulcerative colitis (UC) is a chronic, idiopathic form of inflammatory bowel disease (IBD) characterized by continuous mucosal inflammation that variably extends from the rectum throughout the colon [1]. Its incidence is increasing globally, with an estimated prevalence affecting approximately 5 million individuals worldwide [2]. UC is distinguished by a relapsing and remitting clinical course [3], often resulting in significant morbidity for affected individuals. This morbidity is largely due to debilitating symptoms, such as persistent diarrhea, abdominal pain, rectal bleeding, and fatigue, which not only compromise physical health but also severely impact patients’ quality of life. Additionally, prolonged disease activity increases the risk of long-term complications, such as anemia or colorectal cancer, further underscoring the importance of effective disease management [4,5].

Mesalazine, a well-established therapeutic agent within the salicylate class (ATC code: A07EC02), is widely recognized as the first-line treatment for the acute phase of mild to moderate UC and the maintenance of remission [1,6,7]. Its role in managing IBDs, particularly UC, is supported by over 35 years of clinical experience in Europe, where numerous pharmaceutical formulations containing mesalazine have been extensively tested and marketed. Available in both oral and rectal preparations, mesalazine primarily exerts its effects locally and has been proven to be effective and safe in restoring mucosal integrity and managing inflammation in UC patients.

The therapeutic activity of mesalazine is linked to the modulation of key inflammatory pathways, including inhibition of prostaglandin synthesis [8], inhibition of NF-κB activity, and reduced production of cytokines such as TNF-α and IL-1 [9]. Additionally, mesalazine acts as a ligand for peroxisome proliferator-activated receptor-γ (PPAR-γ), a nuclear receptor involved in regulating intestinal tissue homeostasis [10,11]. Its action on inflammatory mediators, alongside its ability to stabilize the intestinal epithelial barrier and decrease oxidative stress [12], highlights its capacity to reduce immune cell recruitment, epithelial damage, and overall inflammation in UC [10].

The efficacy of mesalazine treatment is closely linked to patient adherence to prescribed medication regimens. However, adherence rates have been reported to be only 40–60%, based on self-reports and urinary drug measurements in patients receiving mesalazine therapy [13,14]. Non-adherence has been associated with a fivefold increase in the risk of relapse, as well as higher rates of hospitalization, surgical interventions, and overall healthcare costs [15,16].

The standard posology of mesalazine for mild to moderate ulcerative colitis in adults ranges from 1500 mg/day to 4800 mg/day, depending on the severity of the flare, the extent of colonic involvement, and whether the treatment is intended for induction or maintenance of remission [17,18,19,20]. Mesalazine can be administered either as a single daily dose or in divided doses, with both regimens demonstrating comparable efficacy [21]. However, patient adherence tends to improve with a single-dose regimen [22,23]. Consequently, the development of a higher mesalazine strength formulation of 1500 mg in gastro-resistant tablets provides an alternative therapeutic option that may reduce the frequency of administration and further enhance patient compliance. From a biopharmaceutical perspective, mesalazine is a BCS Class IV drug, with low solubility and permeability [24]. For gastro-resistant formulations, in vitro dissolution similarity does not reliably predict in vivo absorption, which is influenced by intestinal metabolism and variable gastrointestinal conditions. Moreover, as mesalazine acts locally, systemic exposure mainly reflects comparative safety. Therefore, bioequivalence studies are necessary to assess in vivo performance, even when dissolution profiles are similar [25]. Based on this rationale, the clinical trial presented in this manuscript was designed to compare the relative bioavailability of a single 1500 mg mesalazine gastro-resistant tablet (Test) to that of three 500 mg Claversal^®^ gastro-resistant tablets (Reference). If bioequivalence is demonstrated, it could support the approval of this new higher-strength formulation, potentially providing a preferred therapeutic alternative for patients and enhancing treatment adherence.

## 2. Results

### 2.1. Study Subjects’ Disposition

**Figure 1:**
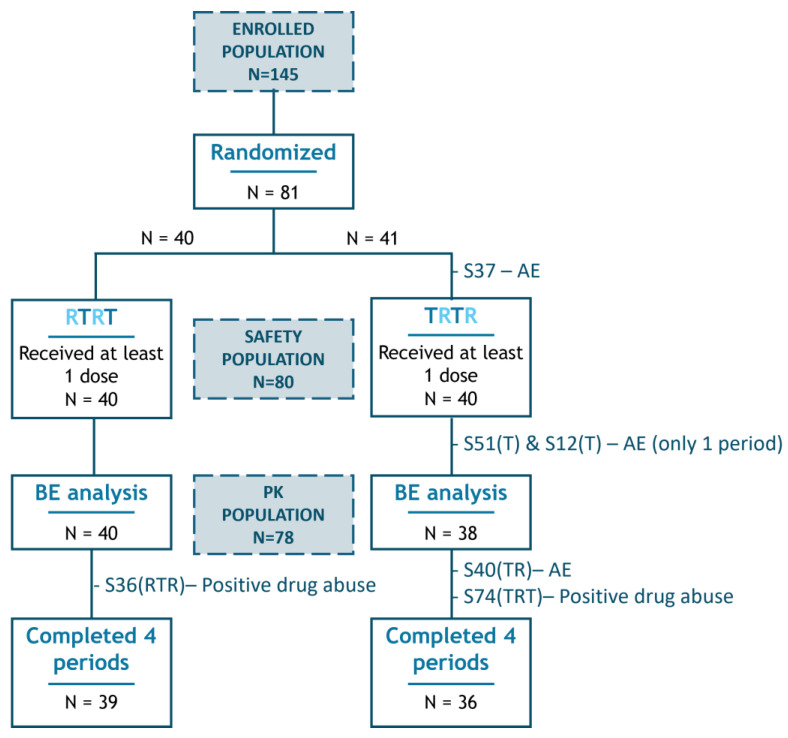
Study subjects’ disposition. Representation of the flow of patients throughout the study and those included in the different populations (indicated by blue-shaded squares). Dropouts are identified by their subject number (SXX), with the completed test (T) or reference (R) periods prior to withdrawal shown in brackets, followed by the primary reason for exclusion. BE, bioequivalence; AE, adverse event.

A total of 145 male and female subjects signed the informed consent form and were enrolled. Per protocol, 80 healthy volunteers were statistically determined to be the optimal study size. As a consequence of a participant withdrawing from the study, due to an Adverse Event prior to study drug administration and being replaced, a total of 81 subjects were randomized, with 80 ultimately receiving the first period of test or reference mesalazine (Figure 1).

All eighty participants who received at least one dose of study medication were assessed for safety, forming the safety population. Only those who completed at least one test and one reference period (i.e., had data for comparison of both medications) and had not experienced any major protocol deviation that might alter the PK evaluation were included in the statistical comparison of bioavailability of both mesalazine formulations and were considered the pharmacokinetic (PK) population (*n* = 78 subjects).

### 2.2. Demographics and Baseline Characteristics

Within the safety population (*n* = 80), 53.75% were male, and 46.25% were female, with a mean age of 27.68 years and an average BMI of 24.88 kg/m^2^. The mean weight and height were 71.76 kg and 169.6 cm, respectively. Regarding racial demographics, 68 participants were categorized as other (mixed), 10 as Caucasian, and 2 as Black. The principal demographic characteristics of the safety population are summarized in Table 1.

Additionally, all data obtained from clinical histories and physical examinations confirmed the compatibility of volunteers with a healthy status. The baseline analytical data of the volunteers fell within normal ranges, with a few cases showing minor deviations that were assessed by the investigator as not clinically significant.

### 2.3. Pharmacokinetic Characterization

**Figure 2:**
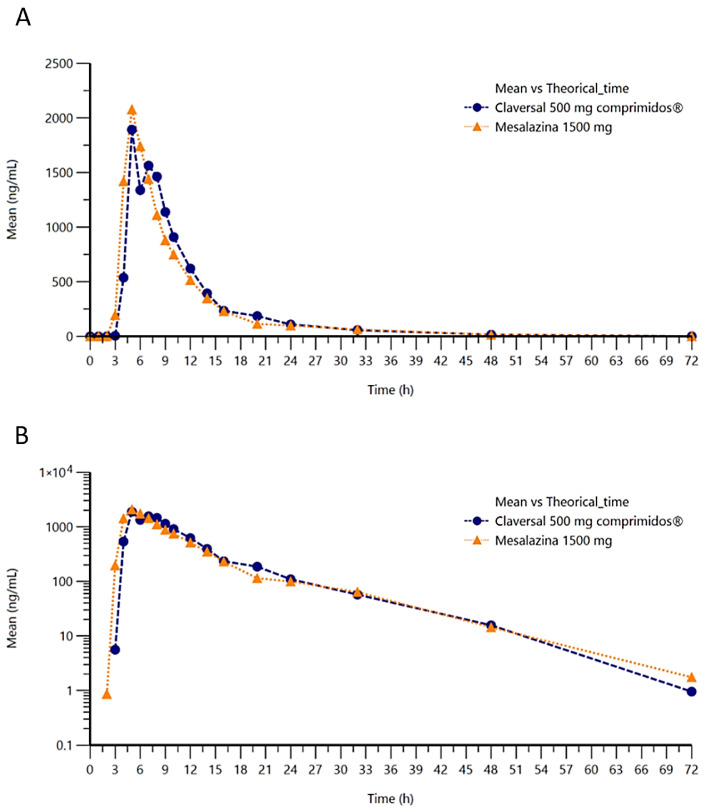
Plot of mean concentration of mesalazine versus time in linear (**A**) and semilogarithmic (**B**) scales.

The concentration of mesalazine was determined at various timepoints over a 72-h period following single-dose administration of 1 tablet of mesalazine 1500 mg (T) or three tablets of Claversal^®^ 500 mg (R). Representation of the concentration–time in linear–linear or logarithmic–linear scales (Figure 2A,B) show similar curves for both formulations of mesalazine.

To further characterize the systemic bioavailability of mesalazine following administration of 3 tablets of Claversal^®^ 500 mg or a single 1500 mg mesalazine gastro-resistant tablet, the pharmacokinetic parameters defining the rate and extent of absorption and elimination were estimated (Table 2).

Elimination rates (Ke) were also similar, at 0.14 ± 0.14 L/h and 0.13 ± 0.08 L/h for the test and reference medications, respectively, and mean elimination half-lives (t_1/2_) of 8.65 ± 8.50 and 9.79 ± 10.43 h for test and reference, respectively. The similar absorption and excretion dynamics resulted in comparable mean residence times of 13.20 ± 7.16 h and 14.50 ± 7.92 h for the test and reference formulations of mesalazine.

### 2.4. Bioequivalence Analysis

To compare the rate and extent of absorption of the highly variable drug (HVD) mesalazine following single doses of 1500 mg of the test and the reference formulations, the parameters AUC_0–∞_, AUC_0–t_, AUC_8–48h_, and C_max_ were modeled and analyzed using a non-compartmental ANOVA test. The geometric mean test/reference ratios and the 90% confidence intervals (CIs) for the mentioned parameters are shown in Table 3.

Following the approach established by EMA guidelines for the evaluation of bioequivalence of HVD and of modified release formulations, bioequivalence acceptance limits for parameters C_max_ and AUC_8–48h_ scaled by the intra-subject coefficients of variation of the reference product were 69.84–143.19% for C_max_ and 74.62–134.02% for AUC_8–48h_. The 90% confidence intervals for the ratio of the population geometric means (mesalazine 1500 mg tablet/Claversal^®^ 500 mg 3 tablets) for both parameters were within these predefined acceptance intervals. Additionally, the point estimates of the geometric mean ratios for C_max_ and AUC_8–48h_ were contained within the narrower 80.00–125.00% range, demonstrating bioequivalence in these parameters. The comparison of the systemic exposure to the drug over time of both mesalazine formulations was also consistent with bioequivalence since the 90% confidence intervals for the ratio for AUC_0–∞_ and AUC_0–t_ were within the acceptance range 80.00–125.00% (Table 3). The statistical power of the analysis was 87.38% for C_max_ and 99.98% for AUC_0–t_, confirming that the sample size was sufficient to ensure adequate power for the bioequivalence assessment.

### 2.5. Safety Results

Eighty participants received at least one dose of each study drug and were included in the safety analyses. A total of seventy-five participants completed the four periods of study treatment, receiving single doses of the test formulation twice and the reference formulation twice. Participants who did not complete all four study periods are represented in Figure 1, with the periods completed prior to withdrawal indicated in brackets. In total, there were 154 exposures to the reference product and 156 exposures to the test product, resulting in a combined total of 310 exposures.

Overall, 51 adverse events (AE; Table 4) were reported among 38 of the 80 participants included in the safety population. None of these events was of severe intensity, nor did any qualify as a serious adverse event (SAE). All events were resolved by the end of the trial. Eight adverse events were related to the study drug, with five occurring after exposure to the reference formulation (3.21% of the exposures to reference) and three after exposure to the test formulation (1.95% of the exposures to test; Table 4).

The reported AEs associated with the test product included flatulence and rash (Table 5). A mild, self-limited rash occurring after administration of the test product (2 subjects) or the reference product (1 subject) was the only AE that led to treatment discontinuation during the study. No deaths or pregnancies were reported. To summarize, the overall incidence of adverse events and the safety profiles were comparable between mesalazine 1500 mg tablets and Claversal^®^ 500 mg (three tablets totaling 1500 mg).

## 3. Discussion

Mesalazine is a well-established medication widely used as a first-line therapeutic option for mild to moderate UC. It has been extensively prescribed for over three decades, and its efficacy and safety are well-documented [7]. Effective long-term treatment with mesalazine offers several advantages, including minimizing the need for more expensive and potentially harmful medications like corticosteroids and biologic therapies [26]. It also contributes to enhanced quality of life, lowers the likelihood of requiring a colectomy in the future, and reduces the risk of colorectal cancer [26].

The mesalazine dosage regimen depends on various factors, such as disease severity, disease phase (relapse or maintenance), patient characteristics, and concurrent medications. The recommended daily dose ranges from 1500 mg to 4800 mg. Whether administered as a single dose or divided into multiple doses throughout the day, its efficiency has been demonstrated to be equivalent [21]. However, adherence to the treatment regimen is a key determinant of the clinical outcome. Kane and colleagues demonstrated in their study involving 99 patients with UC in remission for at least six months that non-adherent patients were more than five times as likely to experience disease recurrence compared to adherent patients. Furthermore, evidence from a study by Frøslie et al. [27], which tracked 740 patients newly diagnosed with UC or Crohn’s disease over a period of five years, highlights that maintained remission strongly correlated with reduced inflammation, lower dependency on corticosteroids, and a decreased likelihood of requiring colectomy in the future. Consequently, improved adherence not only sustains remission but also reduces the risk of hospitalization, surgery, and long-term complications associated with uncontrolled disease progression. This is also supported by the meta-analysis performed by Velayos et al. [28], demonstrating a correlation between mesalazine sustained treatment and lower incidence of colon cancer and dysplasia in UC patients.

Currently, mesalazine is available in various formulations, including granules and tablets, with no significant differences in remission rates observed among these oral formats [21,29]. Additionally, as the efficacy remains consistent whether administered as a single dose or divided doses throughout the day, reducing the number of tablets offers a notable clinical advantage in promoting treatment adherence, especially among the increasing population of polymedicated patients. Scientific studies further support this improved adherence, demonstrating higher compliance rates with simplified dosing regimens.

Mesalazine gastro-resistant tablets are available in 500 mg and 1000 mg formulations. To address the challenge of pill burden in the treatment of ulcerative colitis, FAES FARMA has developed a new 1500 mg formulation. This higher-strength gastro-resistant tablet is designed to maintain the established efficacy and safety profile of mesalazine, while providing a more convenient option for subjects with UC [22].

The current study showed that the test formulation of 1500 mg mesalazine gastro-resistant tablets is bioequivalent to three tablets of the established reference formulation of Claversal^®^ 500 mg (total dose 1500 mg). The 90% confidence intervals of the test to reference products for AUC_0–t_ and AUC_0–∞_ fell within the standard bioequivalence range of 80.00% to 125.00%. Additionally, the 90% confidence intervals for C_max_ and AUC_8–48h_ fell within the bioequivalence ranges scaled by coefficient of variation, as adapted to HVD and modified release forms, respectively, confirming comparable performance between the two formulations. It is noteworthy that the confidence intervals for AUC_8–48h_ and C_max_ do not include 100%. This observation is consistent with the known high intra-subject variability of mesalazine and does not undermine the overall conclusion of bioequivalence.

Secondary pharmacokinetic parameters, including t_max_, t_1/2_, K_e_, CL, and MRT, were also consistent across both formulations. The median t_max_ was 5 h (range 3–14 h) for mesalazine 1500 mg tablets and 7 h (range 4–20 h) for Claversal^®^ 500 mg (3 tablets, total 1500 mg). This 2-h difference in median t_max_ is not considered clinically relevant, as mesalazine’s therapeutic effect depends on its local action in the small and large bowel rather than the timing of peak plasma concentration. Notably, both mesalazine formulations were well tolerated, with no deaths or serious AEs being reported.

Previously reported pharmacokinetic values of mesalazine vary widely across studies depending on formulation type, dose regimen, and prandial state [25,30,31]. Nevertheless, the high inter- and intra-subject variability of mesalazine kinetics is well documented and largely driven by formulation-dependent release and gastrointestinal transit [32]. Given the known high variability of mesalazine, it is expected that absolute PK values may differ between independent studies; what matters for regulatory decision-making is whether test and reference products are equivalent under the same conditions, which is the focus of the present analysis. Regarding safety, our finding of a low frequency of mild adverse events (with no serious or severe events) is consistent with the safety profile described in previous clinical studies of mesalazine formulations [33]. No new safety signals emerged, and the incidence and pattern of treatment-emergent adverse events were within previously reported ranges.

The strength of this study lies in its large sample size, which provides robust evidence that the higher-strength formulation of 1500 mg mesalazine gastro-resistant tablets is bioequivalent to the original formulation, with comparable pharmacokinetic and safety profiles. Additionally, the new formulation offers a clinical relevance strategy to reduce pill burden, potentially improving patient compliance and treatment efficacy. However, a notable limitation is that the 1500 mg tablet may not be suitable for individuals who have difficulty swallowing larger pills. Furthermore, as the study was specifically designed to evaluate bioequivalence, it included only healthy individuals without comorbidities or concomitant medications, in alignment with EMA guidelines. As a result, diversity among participants was not prioritized, and the study was conducted at a single site.

In conclusion, the pharmacokinetic profiles of mesalazine gastro-resistant tablets were thoroughly characterized for both the 1500 mg test tablet and the three Claversal^®^ 500 mg reference tablets. Both formulations demonstrated similar safety profiles in healthy subjects under fasting conditions. These findings confirm that the 1500 mg mesalazine gastro-resistant tablets are bioequivalent to three tablets of the 500 mg Claversal^®^ tablets (total dose 1500 mg), with no expected differences in therapeutic outcomes. The new higher-strength formulation offers a significant advantage by reducing the daily tablet burden for UC patients, potentially improving adherence to the prescribed regimen, and optimizing treatment efficacy.

## 4. Materials and Methods

### 4.1. Trial Design

This phase I open-label clinical trial was designed as a randomized, two-sequence, four-period, crossover replicate study following EMA guidelines for evaluating bioequivalence for highly variable drugs such as mesalazine [34]. The primary objective was to compare the relative bioavailability under fasting conditions of the test formulation of mesalazine 1500 mg gastro-resistant tablets compared with three reference Claversal^®^ 500 mg gastro-resistant tablets, both manufactured by Faes Farma S.A. (Leioa, Spain). The secondary objective was to evaluate the safety and tolerability of the two formulations.

With this aim, single doses of 1500 mg of mesalazine were administered at each of the four periods as one test tablet of 1500 mg (T) or three tablets of 500 mg of the reference product Claversal^®^ (R). To ensure standardization across all four study periods, participants were admitted to the Clinical Trials Unit on the evening prior to each dosing day. The investigational products were administered orally with 240 mL of water after a fasting period of at least 8 h, under the direct supervision of study personnel. As the half-life of mesalazine is around 12 h, sampling was extended up to 72 h post-dose. A total of 19 samples were collected in each period. A washout period of at least 7 days (more than 10 half-lives) before each dose administration was established to ensure that mesalazine plasma concentrations were undetectable at the beginning of the following treatment period, avoiding the risk of a carry-over effect.

### 4.2. Trial Population

Prior to the first admission day, all participants had to give their written informed consent to participate in the study. The main inclusion criteria were healthy male or non-pregnant/breastfeeding female aged 18 to 35 years, with no clinically significant organic or psychological conditions. Participants were required to have no relevant abnormalities in their medical history or physical examination, and their vital signs and electrocardiographic readings had to fall within normal ranges, as determined by current clinical practice guidelines and the investigator’s assessment. Participants were allocated in a balanced ratio to treatment sequences TRTR or RTRT. Randomization was performed in 10 blocks of 8 subjects.

### 4.3. Pharmacokinetic Endpoints

The pharmacokinetic analysis and the comparison of the bioavailability of the two formulations were carried out by assessing the pharmacokinetic parameters that describe the rate and extent of serum drug exposure: the maximum concentration (C_max_) and the area under the curve (AUC_0–t_, AUC_8–48h_, and AUC_0–∞_), calculated from plasma concentrations of mesalazine. The partial AUC between 8 and 48 h (AUC_8–48h_) was used to characterize the delayed release and regional absorption phase of mesalazine and to minimize early variability related to gastric emptying and lag time. This time interval follows the Food and Drug Administration (FDA) product-specific guidance for mesalamine delayed-release products, which recommends assessing bioequivalence based on AUC_8–48h_, AUC_0–t_ and C_max_. Selection of pharmacokinetic parameters was therefore based on these FDA recommendations and the EMA guideline on the pharmacokinetic and clinical evaluation of modified release dosage forms [35,36].

As secondary pharmacokinetic variables, t_max_ was also obtained from plasma concentrations, and the elimination constant (K_e_), the volume of distribution (V_d_), half-life of elimination (t_1/2_), clearance (CL), and mean residence time (MRT) were calculated.

### 4.4. Safety Endpoints

For the safety and tolerability analysis, the following parameters were assessed: incidence of adverse events/untoward events (AEs/UEs), medical history, concomitant medication, physical examinations, vital signs, 12-lead ECG, and clinical laboratory tests (hemogram, coagulation, biochemistry, and urine analysis).

### 4.5. Statistical Analysis

The pharmacokinetic and statistical analyses were conducted at Hospital Universitario de La Princesa, Clinical Pharmacology Department, with Phoenix^®^ WinNonlin^®^ version 8.4 (Certara USA, Inc., Princeton, NJ, USA).

For analysis of bioequivalence, the main pharmacokinetic endpoints: AUC_0–t_, AUC_8–48h_, AUC_0–∞_, and C_max_ were modeled and analyzed using non-compartmental analysis of variance (ANOVA). The confidence interval (CI) for the difference between test and reference formulations on the log-transformed scale was obtained from this ANOVA model and subsequently, backtransformed to obtain the desired CI for the ratio test/reference on the original scale. The ANOVA considered 4 factors: sequence, subjects nested in sequence, period, and treatment.

To calculate the pharmacokinetic parameters, a non-compartment analysis (NCA) based on real sampling times was used. AUC_0–t_ was calculated using the linear trapezoidal method between the first and the last quantifiable concentrations. AUC_8–48h_ was determined using the same method, considering only the concentrations within the 8 to 48 h interval post-dose. AUC_0–∞_ was obtained by adding AUC_0–t_ and the extrapolated area from the last quantifiable concentration to infinity (C_last_/λz), where λz was estimated by linear regression of log-transformed concentrations in the terminal phase, selecting the model with the highest adjusted R^2^, as per WinNonlin’s standard procedure. C_max_ and T_max_ were obtained directly from the observed plasma concentration–time data.

According to the Guideline on the Investigation of Bioequivalence [34], to determine bioequivalence, the 90% confidence interval of AUC and C_max_ parameters for the ratio of the test and reference products should be contained within the acceptance interval of 80.00–125.00%. Additionally, for highly variable drugs such as mesalazine, where within-subject variability for PK parameters exceeds 30%, the bioequivalence acceptance interval for C_max_ may be scaled by the within-subject coefficient of variation (CV) of the reference formulation to a maximum range of 69.84–143.19%. In accordance with EMA Guideline on the pharmacokinetic and clinical evaluation of modified release dosage forms [37], 90% CI acceptance limits could be widened for partial-AUC (AUC_8–48h_) following a similar approach to that for C_max_. As established by the guideline, “The extent of the widening is defined based upon the within-subject variability seen in the bioequivalence study using scaled-average-bioequivalence according to [U, L] = exp [±k·sWR], where *U* is the upper limit of the acceptance range, *L* is the lower limit of the acceptance range, *k* is the regulatory constant set to 0.760 and *sWR* is the within-subject standard deviation of the log-transformed values of C_max_ of the reference product.” Within-subject variability was calculated using the formula $CV\left(\%\right)=100\sqrt{e\;{sWR}^{2}-1}.$

#### Sample Size Estimation

The sample size calculation was performed using the nQuery (v.9.3.1.0) module: “Equivalence Higher-Order Crossover Design for Two Means using Ratios [Four Periods (2 × 4)]” and based on the results obtained in a previous pilot study that shown an intra-subject CV for the reference product (3 tablets of 500 mg) of 50.31% and 97.16% for AUC_0–t_ and C_max_, respectively. Given the acceptance bioequivalence ranges by the EMA guidelines and assuming a true ratio of the geometric means (GMR) of 0.95 and 0.90 for AUC_0–t_ and C_max_, respectively, a total of 72 randomized subjects leads to a test power of 90% and 80% for AUC_0–t_ and C_max_, respectively. Considering sample size calculation and assuming a withdrawal rate of no more than 10% during the study, the inclusion of a total of 80 subjects was deemed appropriate.

### 4.6. Additional Information

The present pharmacokinetic study was conducted at a single site (Clinical Trials Unit, Hospital Universitario de La Princesa, Madrid, Spain) with the registered clinical study identification EudraCT 2021-005439-21. The protocol and informed consent forms were reviewed by the Independent Ethics Committee on Clinical Research (IECCR) of the Hospital “La Paz”, and the date of approval was 29 March 2023. The study was also approved by the AEMPS on 10 April 2023.

The study was conducted in accordance with current Spanish and European regulations [38], and with the ICH guidelines for Good Clinical Practice ICH E6 (R2) and performed according to the Revised Declaration of Helsinki. The first participant was included on 12 February 2024, and the last participant completed the follow-up visit on 3 April 2024.

The study was open for investigators and participants, but blinded for bioanalytical personnel. Plasma concentrations of mesalazine were determined using a validated liquid chromatography–tandem mass spectrometry (LC-MS/MS) method. Analyses were conducted under good laboratory practice at Laboratorios Anapharm Europe S.L.U., Barcelona, Spain.

Safety determinations of hemogram, coagulation, biochemistry, and urine analysis were also performed in an external laboratory, Laboratorio Eurofins Megalab, Madrid, Spain.

## Figures and Tables

**Table 1:** Main demographic characteristics of the safety population (*n* = 80).

Gender	Male	43 (53.75%)
	Female	37 (46.25%)
Race	Black	2
	Caucasian	10
	Other (Mixed)	68
Age (years)	Mean	27.68
	SD	4.51
	Min	18
	Max	35
Height (cm)	Mean	169.60
	SD	8.96
	Min	155.00
	Max	197.00
Weight (kg)	Mean	71.76
	SD	10.65
	Min	50.60
	Max	94.40
BMI (kg/m^2^)	Mean	24.88
	SD	2.63
	Min	19.13
	Max	29.91

**Table 2:** Pharmacokinetic characterization of 1 tablet of mesalazine 1500 mg test formulation and 3 gastro-resistant tablets of Claversal 500 mg reference formulation in the PK population (*n* = 78).

	Mesalazine 1500 mg: Test		Claversal^®^ 500 mg (3 Tablets): Reference	
	Mean	SD	Mean	SD
AUC_0–∞_ (h·ng/mL)	14,821.18	6147.91	14,633.45	6294.23
AUC_0–t_ (h·ng/mL)	14,114.70	6137.78	13,959.30	6675.30
AUC_8–48_ (h·ng/mL)	6858.25	3616.01	8139.50	5311.66
Ke (1/h)	0.14	0.14	0.13	0.08
C_max_ (ng/mL)	3333.07	2551.18	3138.65	2775.52
T_1/2_ (h)	8.65	8.50	9.79	10.43
Vd (L)	1435.37	1354.13	1637.71	161.84
Cl (L/h)	121.89	59.02	130.86	90.63
MRT (h)	13.20	7.16	14.50	7.92
T_max_ (h) mean	5.81	1.91	6.96	2.22
T_max_ (h) median (range)	5.00 (3.00–14.00)		7.00 (4.00–20.00)	

**Table 3:** Main results of the bioequivalence analysis in the pharmacokinetic population (*n* = 78). GMR, geometric mean test/reference ratio; BE range, bioequivalence acceptance range based on EMA guidelines; CI, confidence interval; ISCV, intra-subject coefficient of variation.

Parameter	GMR	Lower CI Limit	Upper CI Limit	BE Range	ISCV%
AUC_0–∞_	102.51	95.85	109.63	80.00–125.00%	31.99
AUC_0–t_	103.36	96.40	110.83	80.00–125.00%	36.65
AUC_8–48h_	84.49	78.24	91.24	74.62–134.02%	43.68
C_max_	114.24	100.15	130.32	69.84–143.19%	80.62

**Table 4:** Summary of adverse events in the safety population (*n* = 80 subjects). Data are presented as the number of adverse events (AEs) and, in parentheses, as the percentage of treatment exposures (*E*).

	Test (*E* = 156)	Reference (*E* = 154)	Total (*E* = 310)
AEs reported	29 (18.59%)	22 (14.29%)	51 (16.45%)
Related AEs	3 (1.92%)	5 (3.25%)	8 (2.58%)
AEs by severity:			
Mild	27 (17.31%)	19 (12.34%)	46 (14.84%)
Moderate	2 (1.28%)	3 (1.95%)	5 (1.61%)
Severe	0 (0.00%)	0 (0.00%)	0 (0.00%)
SAEs reported	0 (0.00%)	0 (0.00%)	0 (0.00%)

*E*, number of exposures to treatment.

**Table 5:** Frequency of related adverse events by System Organ Class and Preferred Term (MedDRA dictionary v27.0). Data are presented as the number of adverse events (AEs) and, in parentheses, as the percentage of treatment exposures (*E*).

System Organ Class (SOC) Preferred Term (PT)	Test (*E =* 156)	Reference (*E* = 154)	Total (*E* = 310)
Gastrointestinal disorders:			
Flatulence	1 (0.64%)	0 (0.00%)	1 (0.32%)
Diarrhoea	0 (0.00%)	2 (1.30%)	2 (0.65%)
Skin and subcutaneous tissue disorders:			
Rash	2 (1.28%)	1 (0.65%)	3 (0.97%)
Nervous system disorders:			
Headache	0 (0.00%)	2 (1.30%)	2 (0.65%)

*E*, number of exposures to treatment.

## Data Availability

In alignment with ethical standards and best practices, we commit to sharing deidentified data from this trial. Data access will be provided to qualified researchers affiliated with academic or research institutions. Interested researchers must submit a research proposal outlining objectives and methodology to clinical_rd@faes.es for sponsor assessment.
